# Vesicular monoamine transporter (VMAT) regional expression and roles in pathological conditions

**DOI:** 10.1016/j.heliyon.2023.e22413

**Published:** 2023-11-15

**Authors:** Malik Alwindi, Ariege Bizanti

**Affiliations:** a St George's University Hospital, London SW17 0QT, United Kingdom; b Burnett School of Biomedical Sciences, College of Medicine, University of Central Florida, Orlando, FL 32816, USA

**Keywords:** Vesicular monoamine transporter, Catecholamine, Dopamine, Norepinephrine, Brain

## Abstract

Vesicular monoamine transporters (VMATs) are key regulators of neurotransmitter release responsible for controlling numerous physiological, cognitive, emotional, and behavioral functions. They represent important therapeutic targets for numerous pathological conditions. There are two isoforms of VMAT transporter proteins that function as secondary active transporters into the vesicle for storage and release via exocytosis: VMAT1 (SLC18A1) and VMAT2 (SLC18A2) which differ in their function, quantity, and regional expression. VMAT2 has gained considerable interest as a therapeutic target and diagnostic marker. Inhibitors of VMAT2 have been used as an effective therapy for a range of pathological conditions. Additionally, the functionality and phenotypic classification of classical and nonclassical catecholaminergic neurons are identified by the presence of VMAT2 in catecholaminergic neurons. Dysregulation of VMAT2 is also implicated in many neuropsychiatric diseases. Despite the complex role of VMAT2, many aspects of its function remain unclear. Therefore, our aim is to expand our knowledge of the role of VMAT with a special focus on VMAT2 in different systems and cellular pathways which may potentially facilitate development of novel, more specific therapeutic targets. The current review provides a summary demonstrating the mechanism of action of VMAT, its functional role, and its contribution to disease progression and utilization as therapeutic targets.

## 1 Introduction

Vesicular Monoamine Transporters (VMATs) are integral proteins located in the membranes of synaptic vesicles, and they are responsible for the packaging of neurotransmitters such as dopamine (DA), serotonin (5HT), norepinephrine (NE), and epinephrine into synaptic vesicles [[1], [2], [3], [4], [5]]. Although the major role of VMATs is to sequester neurotransmitters within vesicles, they are also capable of translocating toxins away from their cytosolic sites of action. Traditionally, it was believed that there is only one vesicular transport of monoamines in the adrenal gland, brain, and other tissues such as mast cells and platelets [[6], [7], [8]]. However, molecular cloning has identified two vesicular transporters for monoamines. Although the predicted proteins are closely related in sequence, they show a range of differences in their physiological and pharmacological properties.

VMAT1, formerly known as the chromaffin granule amine transporter, is present in extraneural tissues, such as the chromaffin cells of the adrenal medulla and the endocrine and paracrine cells of the GI tract, whereas neurons of the central, peripheral, and enteric nervous systems express VMAT2, formerly known as the synaptic vesicular monoamine transporter, exclusively [9]. VMAT2 distinguishes itself as one of the few molecules capable of precisely identifying and transporting all biogenic amine neurotransmitters(DA, 5HT, NE, epinephrine and histamine)[1]. The complex role of VMAT as a transporter vital for life and its use in diagnostic tools and as a potential therapeutic target emphasize its importance and the need to study this protein in depth. This review will highlight VMATs functional role, expression, species differences, and its role in normal and pathological conditions and potential therapeutic target.

### 1.1 History

The field of monoamine transporters was initiated in the late 1950s by the Nobel prize laureate, Julius Axelrod, and his discovery of the norepinephrine transporter. Axelrod's work led to the discovery of other monoamines transporters that eventually became a crucial therapeutic target of many mental disorders [10,11]. Research on serotonin vesicles in 1958 led to the discovery of VMAT. Unknowingly, VMAT has been a therapeutic target in folk medicine for centuries, with the use of Rauwolfia serpentina to treat a wide range of maladies (e.g. insanity, snakebites, fever, and hypertension) [[12], [13], [14],^13^]. The plant has been described in Indian manuscript as early as 1000 BCE, and was named after a German physician Dr Leonhard Rauwolf, who studied this plant while traveling in India [15]. Further research of inhibitors that blocked the uptake of neurotransmitters provided better insight into the possible function of VMAT transporters [16]. It took another decade forthe discovery of genetic tools to identify the DNA, protein and amino acids sequence of VMAT [17,18]. The VMAT was originally found and cloned in rats in 1992 [2] as the principal mediator of monoamine absorption into intracellular vesicles, followed by the discovery of both isoforms (VMAT1 and VMAT2) in 1996 [9].

In 1962, Kirshner showed that reserpine and various so-called “indirectly acting sympathomimetic amines'' drugs like amphetamine, phenylephrine, and ephedrine could block epinephrine uptake into the secretory granule [19]. These experiments established the first molecular link between monoamine uptake into storage vesicles and psychotropic drug action. The study of monoamine uptake into storage granules was followed by an extensive characterization of the remarkable amine-accumulating properties of the vesicular monoamine transporter [20]. More recently, VMAT2 has been the pharmacological target for many neurological diseases and a radiotracer target for positron emission tomography (PET) imaging of neurodegenerative and psychiatric diseases [21].

Recent developments in the use of VMAT2 inhibitors may transform the treatment of some diseases and disorders like Tardive Dyskinesia (TD) [22]. Both Valbenazine (VBZ) and Deutetrabenazine (DBZ) are VMAT inhibitors which have proven effective in treatment of TD [[23], [24], [25]] leading to a significant improvement in TD symptoms, and significantly lower Abnormal Involuntary Movement Scale (AIMS) score [26,27]. Moreover, by using the VMAT2 inhibitor (+)-CYY477, the behavioral effects of amphetamine and methamphetamine in rodents were blocked, indicating that VMAT2 is essential for these drugs to function. VMAT2 inhibition also led to a marked reduction in the ability of amphetamine to alkalize vesicles, suggesting that amphetamines are transported by VMAT into the vesicles [28].

### 1.2 Structure

VMAT1 and VMAT2 are acidic glycoproteins with 12 transmembrane domain proteins and an apparent molecular weight of 70 kDa [1,2]. VMAT1,2 are members of the major facilitator superfamily (MFS), specifically SLC18 which has a broad specificity to many substrates. The initial transfer of monoamine neurotransmitter from the synaptic cleft into the presynaptic neuron is accomplished by highly specific transporters from the non-MFS SL6 transporters including serotonin transporter (SERT), dopamine transporter (DAT) and norepinephrine transporter (NET) [29]. Moreover, low affinity and high capacity transporters including organic cation transporters (OCT1-3, SLC 22A1-3) along with the high affinity and low capacity SLC6 transporter contribute to the equilibrium of monoamines in the central nervous system [[30], [31], [32], [33]].

Both VMAT1 and VMAT2 have been cloned, expressed, and characterized in different species. The crystal structure of VMAT2 is still unknown, and its structure has been studied with mutagenesis and photoaffinity labeling [34]. The most prominent variability between the structure of VMAT1 and VMAT2 is near the N and C terminus and in the glycosylated loops between the transmembrane domains [35]. VMAT1 cDNA clone in humans disclosed an open reading frame (ORF) of 1545 base pairs (protein ∼526 amino acid) and showed only 60 % homology with VMAT2 [9]. Rat VMAT2 consists of 515 amino acids, 12 transmembrane domains, a cytoplasmic N terminus, and a cytoplasmic C terminus [21]. The VMAT2 clone in murine model revealed an ORF of 1551 base pairs encoding 517 amino acids and is highly conserved in mammals, with 92% between rats and humans [36]. In rats and mice, the coding sequence of cDNA for VMAT2 has 70 % homology with VMAT1 whereas VMAT2 for both animal models share 90–96 % homology [36,37]. The N terminus and C terminus of VMAT2 include the principal regulatory domains, as well as an intravesical-linking area between the first and second transmembrane domains and a cytoplasmic linking region between the sixth and seventh transmembrane domains. Both terminal domains are targets for phosphorylation, while the region between the first and second transmembrane domains is a target for glycosylation [34]. Additionally, catecholamines showed 3-fold higher affinity and histamine showed 30 fold higher affinity for VMAT 2 in comparison to VMAT1 [9].

VMAT2 undergoes posttranslational modification (PTM), and phosphorylation and glycosylation are the most often observed VMAT2 PTM [34]. Both the N-terminal and C-terminal cytoplasmic domains of VMAT2 are phosphorylated constitutively. N-terminal phosphorylation is required for the maintenance of VMAT2 monoamine absorption and possibly for stimulant-induced monoamine efflux from synaptic vesicles, but the exact role of phosphorylation is unknown [38,39]. In the absence of C-terminal region phosphorylation by casein kinases, VMAT2 is transported into tiny synaptic vesicles rather than massive dense-core granules [40]. Therefore, C-terminal alteration may have a significant function in vivo in the localization of VMAT2 into monoaminergic synaptic vesicles. In addition to the terminal areas, there are many amino acids with kinase binding motifs on the cytoplasmic connecting region between the transmembrane domains; however, their role in VMAT2 regulation has not yet been investigated [34]. It has been well established that the VMAT mechanism of actions operates by exporting 2H+ from the interior of the vesicle in a stoichiometric manner for every cationic monoamine substrate it imports [41](Knoth et al., 1981). Therefore, VMAT are considered antiporters as they utilize the efflux of H+ to uptake monoamine against their concentration gradient (Fig. 2). The active transport of cytosolic monoamine into storage vesicles against a concentration gradient is facilitated by a vesicular H + ATPase located in the granule membrane. This process is driven by PH and electrochemical gradients generated by the V-ATPase [29](Ann and Gasnier, 2014).

Furthermore, few direct interactions between VMAT2 and proteins have been found. Among these is the creation of a complex between VMAT2, tyrosine hydroxylase (TH), and aromatic amino acid decarboxylase (AADC) [42]. TH and AADC, the rate-limiting and final enzymes in DA production, interact directly with VMAT2 on isolated synaptic vesicles in dopaminergic brain areas [43]. The presence of these complexes shows that DA is spatially confined inside the presynaptic terminal, as DA absorption into synaptic vesicles occurs soon after synthesis via VMAT2. This could be interpreted that trafficking and sorting play a significant role in the formation of VMAT2-TH-AADC complexes, which appear to reside exclusively on a subset of vesicles.

### 1.3 Functional role

**Fig. 1:**
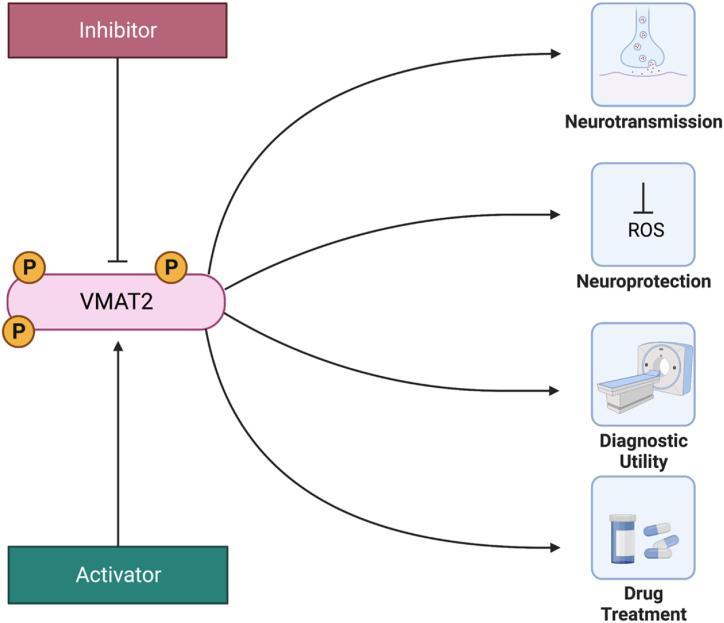
Schematic showing the major functional roles of VMAT2.

While the primary function of VMATs is to sequester neurotransmitters within vesicles (a crucial step in the regulation of neurotransmitter release), they can also translocate toxins away from cytosolic sites of action [38,44]. Both VMAT1 and VMAT2 are expressed in the human adrenal medulla, and may potentially serve a function in peripheral adrenaline release by some psychoactive drugs [45]. In the past several years, human variants of VMAT1 have been linked to susceptibility for schizophrenia and bipolar depression and variants of VMAT2 to schizophrenia and protection from alcohol neurotoxicity [[46], [47], [48]]. For example, in the case of dopamine, the dual role of VMAT2 (neurotransmission and neuroprotection) is combined as it stores DA within vesicles for future transport while preventing it from auto oxidation in the cytoplasm [49]. Furthermore, the harmful effects of exogenous toxins on dopamine neurons, such as MPTP (1-methyl-4-phenyl-1,2,3,6-tetrahydropyridine), can be attenuated by VMAT2 activity which makes it an important pharmacological target [49,50]. The active metabolite of MPTP can be kept within vesicles and prevented from disrupting mitochondrial function and consequently protecting the dopamine neuron. More recently, VMAT2 knockout mice have established the critical role of VMAT2 both in maintaining catecholamine and serotonin levels in the CNS, and monoamine availability for exocytotic release from neurons upon depolarization [51]. An additional potential role of VMAT2 is its involvement in obesity-related hypertension as it has been demonstrated that perivascular adipose tissue stores and transports norepinephrine in a VMAT-dependent manner [52]. Moreover, PET scanning for VMAT2 quantitative assessment is used clinically for early diagnosis and monitoring of the progression of Parkinson's and Alzheimer's diseases and drug addiction. In particular, VMAT2 radiotracer [^11^C]DTBZ and [^18^F]FP-DTBZ radioligands, are being successfully used for human PET studies of neurodegenerative, nigrostriatal DA deficit in Parkinson's disease (PD) and psychiatric diseases [53,54]. Additionally, VMAT2 inhibitors are used to treat dyskinesias caused by neuroleptic drugs or other diseases [55]. For example, tetrabenazine is utilized in the treatment of tardive dyskinesia, a movement disorder that can be caused by prolonged use of antipsychotic medications, and chorea associated with neurodegenerative diseases such as Huntington's disease [56,57]. VMAT2 is also used to identify the functionality of catecholaminergic TH positive neurons. For example, the classical catecholaminergic neurons will express VMAT2 in addition to TH and AADC. Neurons that do not express VMAT2 are sometimes described as non-exocytotic catecholaminergic neurons, even though they express the other two catecholaminergic proteins. The adult mammalian nervous system contains catecholaminergic neurons that might be nonfunctional due to their lack of VMAT2 and consequently inability to transport and store dopamine in the synaptic vesicles [42]. Fig. 1 Shows a schematic of the main functions of VMAT2.

**Fig. 2:**
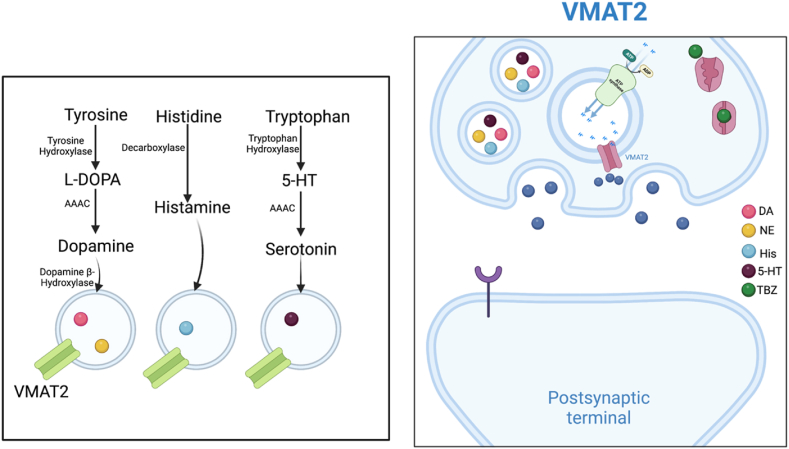
Schematic showing species differences in the location of expression of VMAT.

### 1.4 Species differences in the distribution of VMAT1 and VMAT2

**Fig. 3:**
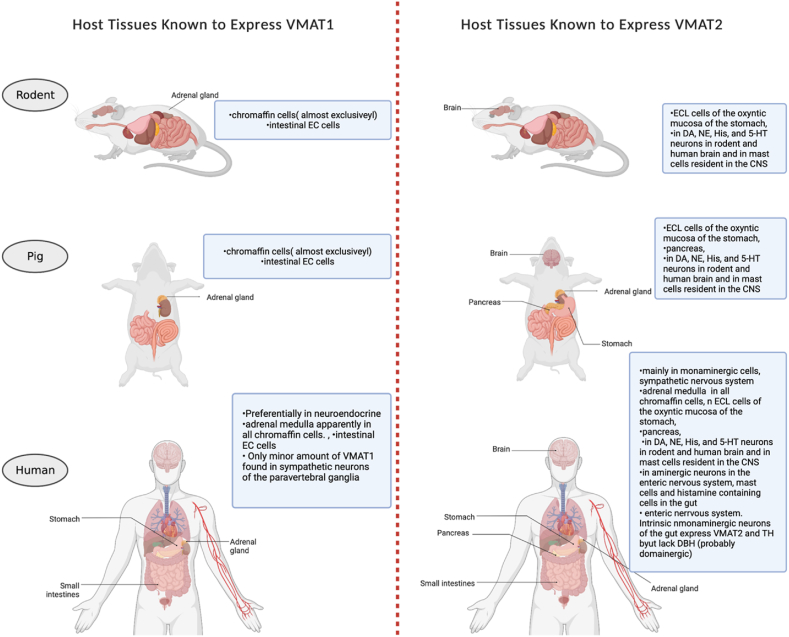
VMAT2 mechanism of transporting monoamines.

Despite the closely related sequence, VMAT1 and VMAT2 have distinct physiologic and pharmacological properties and tissue distribution [9,58]. The distribution of VMAT1 and VMAT2 varies across species and different developmental stages [59]. An understanding of the preferential expression of VMAT1 and VMAT2 and their localization will provide a new foundation for the application of therapies in both fundamental and clinical research of monoamine physiological function and pathological conditions. We summarized the main species differences observed in the literature. Fig. 3 and Table 1 describes the main species and expression differences of these two proteins.

**Table 1:** Comparison of VMAT isoforms.

VMAT1	VMAT2
**In rats**, VMAT 1 expressed in the adrenal gland [60]. Preferentially expressed in neuroendocrine [58,61]	Expressed in the brain [60]. Primarily expressed in the CNS [58,61]
**In bovine**, expressed in adrenal gland and possibly addressed to large secretory vesicles [61]	Expressed in Adrenal gland, major transporter of chromaffin and addressed to small synaptic vesicles [61]
**In humans**, ● VMAT1 is preferentially expressed in large dense vesicles of neuroendocrine cells, such as chromaffin and enterochromaffin cells [58,60]. Both VMAT1 and VMAT2 expressed in chromaffin cells of the adrenal medulla. ● VMAT1 gene SLC 18A1 locus is strongly related to schizophrenia [35]	● VMAT 2 is largely expressed in many monoaminergic cells in the brain, sympathetic nervous system, mast cells, and in histamine-containing cells in the gut [9]. ● VMAT2 plays a role in addiction and psychostimulant induced neurotoxicity [[22], [62]]. ● VMAT2 inhibitor tetrabenazine used for the treatment of chorea [35]. ● VMAT2 used for PET scanning and assessment of Parkinson's and Alzheimer's disease [53,63].

### 1.5 VMAT & pharmacology

VMAT2 has long been considered a promising target for therapeutic interventions. Reserpine, an alkaloid first isolated from Rauwolfia serpentina plant in 1952(58) It has been used for decades as an antihypertensive [64]. It works by irreversibly blocking VMAT2 transport and halts the adrenergic neurotransmission pathway. Additionally**,** VMAT inhibitors are known treatments for movement disorders. Tetrabenazine and deutetrabenazine, two FDA-approved medications for the treatment of chorea associated with Huntington's disease, however, deutetrabenazine has a distinct pharmacokinetic profile with possibly superior safety [65]. VBZDBZ are both VMAT inhibitors that have shown remarkable efficacy in alleviating TD symptoms [[23], [24], [25]]. Furthermore, VMAT2 may be linked to the therapeutic effects of lithium (a common treatment of bipolar disorder) as northern analysis revealed an overall increase (199 ± 27 %) of the neuronal VMAT isoform (VMAT2) in rat brains after a lithium supplemented diet [66]. Amiodarone (an antiarrhythmic drug) is another medication that works via VMAT2 inhibition to prevent the uptake of norepinephrine into the axoplasmic storage vesicle. Furthermore, amiodarone competes specifically with reserpine for binding to VMAT [67]. VMAT2 has also been associated with the mechanism of action of drugs treating affective disorders. For instance, Fluoxetine (SSRI antidepressant) exposure was shown to lead to downregulation of VMAT [68]. Bupropion (NDRI depressant) leads to an increase in vesicular DA uptake associated with redistribution of VMAT2 [69]. Knock out of VMAT2 increased sensitivity to alcohol sedation [70]. The VMATs are also the principal targets of action for a number of psychostimulants and recreational drugs. For example, cocaine led to a rapid increase in VMAT2 ligand binding by altering vesicular monoamine (DA) transport and cytoplasmic DA concentration which cause stimulant-induced changes [71]. I This was supported through experiments involving purified vesicular preparations, specifically striatal synaptosomes. These synaptosomes were subjected to treatment with cocaine or methamphetamine and then homogenized to separate the synaptosomal membrane from the supernatant containing vesicle enriched content [72]. The results of these experiments revealed distinct effects of cocaine and methamphetamine on the distribution of VMAT2 as cocaine treatment caused VMAT2 to relocate from the synaptosomal membrane to a vesicle enriched fraction. On the other hand, methamphetamine treatment led to the redistribution of VMAT2 from vesicles to a non-synaptosomal location as indicated by a decreased VMAT2 in all synaptosomal fractions ([[73], [74], [75], [76], [77],^72^]). In a somewhat similar mechanism to cocaine, Phencyclidine (PCP) (hallucinogenic drug) led to a rapid increase of vesicular dopamine uptake and binding of dihydrotetrabenazine (VMAT2 Ligand) [78]. It is well established that the neurotoxic effects caused by psychostimulant drugs involve their interaction with VMAT2 [62]. This interaction leads to the release of monoamine neurotransmitters from synaptic vesicles into the cytosol, resulting in neurotoxicity. Low VMAT2 expression in rats lead to dopamine related neurotoxicity and nigrostriatal degeneration, while elevated VMAT2 expression provides a protective benefit against methamphetamine neurotoxicity [79]. Many substances that are transported by VMAT2 are also associated with monoaminergic neurotoxicity. For instance, (±)-cis-4,4′-dimethylaminorex (4,4′-DMAR) [62], is a psychoactive substance, led to the inhibition of VMAT2, in both rat PC12 cells and human isoforms. (4,4′-DMAR), the effect was comparable to MDMA (3,4, methylenedioxymethamphetamine), but significantly less than reserpine which could explain the potential long-term neurotoxicity [[80], [81]]. Mephedrone (4-methylmethcathinone, MMC) is an amphetamine like substance and a popular component of party drugs showed ten times less effectives than MDMA inhibiting VMAT2, which may contribute to its lower long-term neurotoxicity [[81], [82], [83]]. High doses of amphetamines have been shown to deplete monoamines in the brain by interfering with VMAT2 and disrupting the storage of neurotransmitters in vesicles. One study suggests that cathinone-derived compounds, such as S-4-MC and S-4-TFMMC, may have reduced neurotoxic potential compared to other substances like S-4-MMC. This reduction in neurotoxicity is attributed to the weaker inhibitory effects of S-4-MC and S-4-TFMMC on VMAT2 [84].

It is worth noting that there is a specificity associated with VMAT2, for example, both haloperidol and clozapine are considered antipsychotic medications but only clozapine lead to an increase in [3H] TBZOH binding in the nucleus accumbens, prefrontal cortex and striatum while haloperidol had no effect on VMAT2 [85]. It is also known that VMAT2 is associated with medications used to treat Parkinson's disease, including pramipexole and apomorphine.

Pramipexole led to a redistribution of VMAT2 immunoreactivity within nerve terminals in the striatum of treated rats [86], whereas apomorphine was shown to lead to a rapid and reversible increase of dopamine in rat purified striatal vesicles. This was associated with a redistribution of VMAT-2 in nerve terminals [87,88]. A summary of the different disease treatments that are associated with VMAT2 is shown in Table 2.

**Table 2:** Diseases with treatments associated with VMAT2 pathway.

Huntington's disease chorea	Tetrabenazine, deutetrabenazine [89].
**Depression**	Fluoxetine; Bupropion [68,69]
**Parkinson's**	Pramipexole, Apomorphine [86,87]
**Bipolar disorder**	Lithium [66].
**Arrhythmia**	Amiodarone [67].
**psychostimulants**	Cocaine, methamphetamine, Phencyclidine [[73], [74], [75], [76], [77]]
**psychosis**	Clozapine [85]
**Tardive Dyskinesia**	Valbenazine, Deutetrabenazine [[23], [24], [25]].

### 1.6 VMAT and clinical diseases

VMAT contributes to many clinical disorders and plays an important role in the pharmacology of many treatment modalities. Based on recent discoveries, neuroradiologists and neuropathologists can now utilize VMAT2 as a target for in vivo imaging and drug development. Here we will discuss the involvement of VMAT in a wide range of diseases and body systems.

### 1.7 VMAT & gastrointestinal (GI) tract

The major monoamine-containing cells of the digestive system consist of neurons of the enteric nervous system, the enterochromaffin (EC) cells of the intestine, and enterochromaffin-like (ECL) cells of the stomach [90,91]. EC cells express only VMAT1 which is hypothesized to be unable to transport Histamine, in contrast, the ECL cells of the stomach express VMAT2, synthesize and accumulate histamine from the diet [59,92]. Histamine secreting ECL cells and serotonin secreting cells are the major cell types in the acid producing stomach, and in contrast to midgut neuroendocrine tumors which produce more serotonin, gastric carcinoids originating from ECL rarely produce any serotonin [92]. These intestinal endocrine cells need a selective uptake of serotonin from the diet, not histamine [38,93]. The inability of VMAT1 to recognize and transport histamine correlates structurally to its lack of binding to tetrabenazine (TBZ), a specific ligand of VMAT2. The specific site responsible forTBZ binding to VMAT2 has been mapped with VMAT1/VMAT2 chimeric proteins [59].

### 1.8 VMAT & Parkinson's

Parkinson's disease (PD) is a devastating neurodegenerative disease and its hallmark is the loss of dopamine neurons [94]. The disease is distinguished by the major symptoms of resting tremor, postural instability, stiffness, and bradykinesia [94,95]. The incidence of PD is positively correlated with age; as it was demonstrated that there is more than 40-fold increase in prevalence of PD between the ages of 55 and 85 [96]. VMAT2 has been suggested as an excellent marker of presynaptic dopaminergic nerve terminals in the striatum of PD patients [16,45,97]. Pathogenic changes in PD are extensive and mainly characterized by the loss of dopaminergic neurons in the substantia nigra pars compacta and loss of striatal innervation. Abnormal dopaminergic tone is connected to multiple neurological disorders, including Parkinson's disease, schizophrenia, and psychoses [98]. Administration of MPTP leads to death of dopaminergic neurons and produces clinical Parkinsonism. Upon entrance to the brain, MPTP is metabolized to 1-Methyl-4-phenylpyridinium (MPP+) which leads to inhibition of respiration and mitochondrial damage by affecting oxidative phosphorylation [99,100]. Since all catecholamines are reactive molecules, the metabolic turnover of DA is also tightly regulated by its synthesis, degradation, and compartmentalization functions of this VMAT [[101], [102]]. Dopamine is sequestered into synaptic vesicles, thereby occupying a unique role by facilitating dopaminergic neurotransmission. The sequestration prevents the deleterious effects of dopamine in the cytosol. Thus, there is a direct association with VMAT2 as the regulation of cellular dopamine homeostasis is directly affected by VMAT2 activity. The distribution of VMAT2 in control and Parkinson's disease in the human brain has been examined in vivo and in vitro using a variety of radiolabeled tetrabenazine analogs [103]. It resembles a known distribution pattern of monoaminergic cell bodies and their projections. In vivo imaging and postmortem binding studies showed significant reductions in VMAT2 immunoreactivity in the caudate, putamen, and nucleus accumbens of PD brains. Interestingly, a gain of function haplotype of VMAT2 was shown to be protective against the development of PD in humans. Despite these data, it is still unclear if the reduction in VMAT2 expression contributes to or causes PD [104]. Further experiments are needed to confirm if there is a causative relationship between VMAT2 depletion and PD. Considering the potential therapeutic role of monoamine transporter, multiple treatment approaches have been utilized to improve monoamine neurotransmitter signaling [105]. Many of these therapies, however, have adverse side effects or lose efficacy due to off-target activities and system feedback. These negative effects are presumably the result of neurotransmitter release and temporally dysregulated absorption. Increasing vesicular packing was shown to improve dopamine neurotransmission without disrupting signaling. A study on mice showed that increased levels of vesicular monoamine transporter exhibit greater dopamine release, enhanced mobility, and protection from a neurotoxic insult associated with Parkinson's disease [44]. The malleability of the dopamine vesicle implies that therapies designed to increase vesicle fullness may have therapeutic value. This pathway would also indicate the potential of increasing the levels of VMAT2 expression in opposing Parkinson's related neurodegeneration [104].

### 1.9 VMAT & movement disorders

Huntington's disease (HD) is a hereditary neurodegenerative disorder that follows an autosomal dominant pattern of inheritance, and it is characterized by a gradual and selective loss of neurons in the affected tissues, particularly in the striatum [106].

One of the main clinical features that leads to HD diagnosis is movement disorders, psychological and cognitive impairments, but there is currently no effective treatment to treat this condition. VMAT2 inhibitors like tetrabenazine and deutetrabenzaine are used to alleviate the movements associated with Huntington's [65]. However, the mechanisms of the therapeutic effect of these inhibitors on HD is still largely unclear. Recently, a study evaluated the therapeutic effect of a new VMAT2-inhibitor called NBI-641449 in the treatment of HD with low toxicity to neurons in the striatum and reducing off target side effects which holds therapeutic potential for the treatment of Huntington's disease [107]. Zebrafish VMAT2 (SLC8A2) mutant strains that were recently developed demonstrated that the absence of Vmat2 increases monoamine turnover and activates genes responsible for producing amine enzymes, such as histidine decarboxylase. Mutants lacking Vmat2 also experience downregulation of Notch1a and pax2a, genes linked to stem cell development and exhibited abnormal locomotion and behavioral phenotypical changes [108,109]. Moreover, Variation in a single nucleotide polymorphism in SLC18A2 gene encoding VMAT2 have been implicated in the occurrence of Tardive Dyskinesia (TD) which could potentially give insight on the specific therapeutic target [110]. VMAT inhibitors are novel medications that have recently been used to treat TD, a movement disorder which can be caused by prolonged use of antipsychotic medications. Both Valbenazine (VBZ) and Deutetrabenazine (DBZ) are VMAT inhibitors which have proven effective in treatment of TD [[23], [24], [25]].

### 1.10 VMAT & cancer

Neuroendocrine tumors can generate hormones, then deposit them in vesicles and secretory granules inside the cancer tissue [111]. In fact, the presence of granules and vesicle proteins' is a sign of neuroendocrine tumor development. Notably, the transport of amines into the vesicles of neuronal and endocrine cells is mediated by VMAT1 and VMAT2 [112]. VMAT2 and VMAT1 are reliable markers for differentiation of gastric endocrine hyperplasia and neoplasia from ECL and EC and potentially valuable markers in categorizing neuroendocrine cancer [113,114]. The relevance of VMAT2 and VMAT1 as prognostic indicators stems from the comparatively poor prognosis of EC carcinoma compared to ECL malignant carcinoma, defined by VMAT2 positive diagnostic result [113,115]. Interestingly, in neuroendocrine carcinoma, the lack of both VMAT2 and VMAT1 may suggest a poor prognosis [113]. The pattern of VMAT1 and VMAT2 variable expression in gastrointestinal endocrine tumors is unique to each tumor type and reflects the neuroendocrine development and genesis of the tumors [112]. The lack of VMAT1 expression and strong expression of VMAT2 characterize pancreatic EC cell tumors and differentiate them from intestinal ones [116]. Also, they can indicate radioisotope therapy's usefulness for some patients, depending on expression pattern [117]. Although human pancreatic beta cells express VMAT2, insulinomas or pancreatic endocrine tumors frequently lose VMAT2 expression. VMAT2 expression is also high in mast cells, leading to the accumulation of serotonin and histamine in variable ratios, and providing possible diagnostic opportunities for mastocytomas [116]. Certain treatments take advantage of the fact that these cancers express VMATs in their cells. Their presence makes it possible to utilize radiolabeled ligands that enter the route leading to the production and storage of catecholamines [114]. In most pheochromocytomas and paragangliomas, both VMAT1 and VMAT2 are significantly expressed, with VMAT1 being more frequent in paragangliomas [118]. Collectively these findings make a strong case for the use of VMAT in diagnostic testing.

### 1.11 VMAT and stress

Stress elicits complex physiological, emotional, and metabolic reactions. Its impacts are notable on various levels, from biochemical indicators in plasma to gene expression of specific enzymes [119]. The sympathetic nervous system and its interaction with the adrenomedullary and catecholaminergic pathways in the brain are directly connected to the effects of stress on the body [120]. Neuronal and nonneuronal adrenergic systems may contribute to stress adaptation and tolerance. Both sympathetic and catecholaminergic neuron varicosities in the brain include cytoplasmic vesicles [121]. These vesicles actively store cytoplasmic catecholamines produced or retrieved by VMATs [122]. It was demonstrated that VMAT2 deficiency causes anxiety-like behaviors in zebrafish ([101,108]). In general, all catecholamines are stronger substrates for VMAT2 than VMAT1 [9]. Contrary to popular belief, vesicular reserves of catecholamines are not in a static condition, inertly awaiting exocytotic release [123]. Instead, they reside in a highly dynamic equilibrium with the surrounding cytoplasm, with passive outward leakage of catecholamines balanced by inward active transport regulated by VMAT [124]. Catecholamines removed by neuronal or extraneuronal uptake are transported into storage vesicles. Alternatively, they are metabolized by monoamine oxidase (MAO) in the cytoplasm of neurons, or by catechol-*O*-methyltransferase (COMT) in nonneuronal cells. Monoamine transporters play an important role in the metabolic and physiological functions of catecholamines [125]. This pathway is significantly stimulated by stress; however, there is little interest in research focused on stress-induced alterations in activity and gene expression of peripheral neuronal catecholamines transporters. This is surprising since VMATs play a critical role in modulating the stress pathway. Eisenhofer et al. have demonstrated that exercise increases the rates of norepinephrine release and absorption while leaving the rate of norepinephrine leakage from storage vesicles unaltered [123]. Taken together, these findings indicate indirectly that VMAT are more activated under stress.

### 1.12 VMAT and depression

As we mentioned above, VMAT accumulates biogenic monoamine neurotransmitters in the storage vesicles of presynaptic neurons in a nonselective manner [22]. According to studies, VMAT2 heterozygous (HET) mice mutants exhibited retarded locomotion and rearing in the open field and an aversion to 1 and 1.5 % sucrose solutions which is a test that indicates anhedonia [126]. Immobility durations were lengthened for VMAT2 heterozygotes during forced swimming, and imipramine restored this behavior. In addition, HET animals exhibited increased immobility in tail suspension, which was mitigated by antidepressants like fluoxetine, reboxetine, and bupropion. Stimulated G-proteins coupled receptors binding suggested that 2-adrenergic receptors in the hippocampus of HET mice were more susceptible to stimulation with a specific adrenergic receptor called UK 14,304 (5-bromo-N-(4,5-dihydro-1-H-imidazole-2-yl)-6-quinoxalinamine) than those of wild-type animals (114). This study showed that VMAT2 heterozygotes have a depressive-like phenotype devoid of anxiety-like behavior. Moreover, it's proposed that a reduction in monoamine neurotransmitters is a potential cause for the wide range of depressive symptoms suggesting that altered activity of vesicular monoamine transporters (VMATs) could contribute to the susceptibility of developing affective disorders [109]. Additionally, research demonstrated that the antidepressant bupropion enhances VMAT2 activity, suggesting its potential involvement in the drug's mechanism of action [127].

Interestingly, in individuals with serious depressive illnesses, men express less VMAT1 whereas females express more VMAT2 (Postmortem) [6]. There is, therefore, a definite need for further studies to delineate VMAT specific roles in different species, sexes, physiological and pathological conditions.

## 2 Conclusion

In this review, we have summarized current, state-of-the-art knowledge about VMAT and its diverse roles in cancer and neuropsychological disorders such as depression or Parkinson's disease. We described differences in the distribution of VMAT1 and VMAT2 across different species, as well as its reaction to various pharmacological treatments. The article highlights the complexity of the topic and emphasizes the need for further research. In conclusion, we find that VMAT2 expression has implications for the functioning catecholamine neurotransmission, its disruption in pathological conditions, stress and drug abuse, and its rescue via gene therapy strategies in neurodegenerative disorders like Parkinson's disease.

## 3 Data availability statement

No data was used for the research described in the article.

## Additional information

No additional information is available for this paper.

## CRediT authorship contribution statement

**Malik Alwindi:** Conceptualization, Writing – original draft, Writing – review & editing. **Ariege Bizanti:** Writing – original draft, Writing – review & editing.

## Declaration of competing interest

The authors declare that they have no known competing financial interests or personal relationships that could have appeared to influence the work reported in this paper.
